# A Clinical Study of Intraoperative Perfusion Chemotherapy With Raltitrexed in Colon Cancer: A Prospective Cohort Study

**DOI:** 10.7759/cureus.58481

**Published:** 2024-04-17

**Authors:** Khan Adnan, Saddam Hussain, Muhammad Amir, Sohail Ahmed, Amna Akbar, Sarosh Khan Jadoon, Sania Khan, Zhang ZiLong, Mohammad Saleem Khan

**Affiliations:** 1 Gastrointestinal Surgery, Yangtze University, Jingzhou, CHN; 2 General Surgery, Zia Darman Hospital, Mardan, PAK; 3 Emergency, Midland Doctors Medical Institute, Muzaffarabad, PAK; 4 Emergency and Accident, District Headquarter (DHQ) Hospital Jhelum, Muzaffarabad, PAK; 5 General Surgery, Combined Military Hospital, Muzaffarabad, PAK; 6 Oncology, Shaukat Khanum Memorial Cancer Hospital and Research Centre, Lahore, PAK; 7 Oncology, Jingzhou Central Hospital, Jingzhou, CHN; 8 Medicine, District Headquarter (DHQ) Teaching Hospital, Kotli, PAK

**Keywords:** adjuvant therapy, chemotherapy, perfusion, raltitrexed, colon cancer

## Abstract

Introduction: Colorectal cancer (CRC) ranks as the second leading cause of cancer-related mortality among women and the third leading cause of cancer-associated mortality among men. Treatment of colon cancer is very crucial for a patient’s survival. In this study, we assessed the reliability, efficacy, and safety of raltitrexed in intraoperative intraperitoneal chemotherapy for colon cancer.

Methodology: A total of 57 patients with clinical stages II and III of colon cancer were included in the study. R0 resection surgery + hyperthermic intraperitoneal chemotherapy (HIPEC) procedure was done with raltitrexed. It was given in a dose of 3 mg/m^2^ in a 0.9% NS injection in a volume of 500 milliliters. Postoperative complications were observed.

Result: The most common postoperative complication was nausea/vomiting, which was seen in 21 out of 57 patients (37%). The second most common complication was fever (18/57). None of the patients died or developed renal toxicity, hepatic toxicity, and intestinal obstruction.

Conclusion: Raltitrexed is a reliable, efficient, and safe drug and can be used in intraoperative intraperitoneal chemotherapy of colon cancer.

## Introduction

Colorectal cancer (CRC) is a serious global health concern. It is the third most common malignant carcinoma diagnosed and the third most prevalent cause of carcinoma-related mortality. In addition, following surgery, about 33% of patients experience recurrence. It is widely acknowledged that free malignant cell implanting in the peritoneum is one of the primary causes of this phenomenon [1].

Oceania and Europe have greater rates of CRC, while Asia and Africa have lower rates. A good lifestyle that includes eating a balanced diet, getting enough exercise, and abstaining from obesity will help prevent CRC. A higher intake of processed or red meat may raise the risk of CRC, whereas fruits, vegetables, and fiber particularly from whole grains and cereals may lower the risk. Risk factors or predisposing circumstances are a family history of the disease, polyps, or a hereditary syndrome (e.g., Lynch syndrome polyposis) [2].

Between 1990 and 2019, the number of CRC incident cases worldwide increased to 2.17 million, while the number of deaths climbed to 1.09 million. From 1990 to 2019, the global age-standardized incidence rate increased from 22.2 per 100,000 to 26.7 per 100,000, while the age-standardized mortality rate decreased from 14.3 per 100,000 to 13.7 per 100,000. A notable increase in incidence rates was noted in younger adults (less than 50 years of age) between 1990 and 2019, especially in nations with a high sociodemographic index (SDI). Globally, in 2019, the leading causes of CRC-related deaths by use of alcohol (9.9%), low-calcium diet (12.9%), smoking (13.3%), and low milk consumption (15.6%) were identified [3].

Globally, there has been evidence of a comparable growth of CRC among younger adults and elderly. By 2030, approximately 15% of CRCs will be diagnosed in younger persons despite general population trends toward aging.

The risk factors in young adults can be obesity, type 2 diabetes, and dyslipidemia acting to trigger the immune response by chronic inflammation mediated by adipocytokines, high concentrations of insulin-like growth factors with impaired insulin receptor activation, stimulation of neoplastic cells with triglycerides, and cholesterol build-up in the membranes of cancer cells [4].

The prognosis for CRC patients is typically poor due to the relative ineffectiveness of standard treatment, such as systemic therapy or surgical excision. Postoperative adjuvant chemotherapy (AC) is now widely acknowledged as a standard treatment for high-risk stage II and stage III CRC patients, although the ideal timing to begin AC remains unknown. If you wait more than eight weeks after surgery to start AC, you are more likely to have a bad outcome. When AC started within eight weeks of surgery, it helped people with CRC more. It was not clear what the difference was in survival gains between AC within five to eight weeks and four weeks. A five- to six-month delay helped patients' prognosis more than those who did not receive AC following surgery [5].

Healthcare workers are becoming aware of the importance of the peritoneal cavity as a metastatic site for a variety of cancers due to advancements in radiological imaging techniques and knowledge. Chemotherapy chemicals delivered intraperitoneally are regarded to be an appealing alternative for surgery or chemotherapy alone since they produce large concentrations in tumor tissues with minimal systemic exposure. The goal of adding hyperthermia to chemotherapy is to increase its anti-tumor effects; this led to the development of heated intraperitoneal chemotherapy (HIPEC), which has been used to treat peritoneal metastases (PMs) for approximately 30 years [6].

PMs are undervalued and associated with a dismal prognosis in CRCs. PMs are difficult to identify during full-body computed tomography (FBCT) tumor staging, even when the secondary localizations of the tumor are obvious in the liver or chest. There is a need to discover treatments for tumor complications, such as obstruction and malignant ascites, remove any macroscopic cancer on the peritoneal surface, and improve median survival. Surgery in conjunction with HIPEC may be a viable therapy option, particularly for younger patients (less than 50 years old) with locally metastasized (pT4) CRC [7]. Treatment of colon cancer is very crucial for a patient’s survival. In this study, we assessed the reliability, efficacy, and safety of raltitrexed in intraoperative intraperitoneal chemotherapy for colon cancer.

## Materials and methods

This study aimed to assess the reliability, efficacy, and safety of raltitrexed in intraoperative peritoneal chemotherapy for colon cancer. This study was conducted at Yangtze University, Central Hospital Jingzhou, China. Patients aged between 18 and 75 years with a diagnosis of CRC confirmed by biopsy and histologic testing of TNM stage II and III were included. The patients were undergoing colorectal R0 resection. The permission for the study was granted by the Ethical Committee Central Hospital Jingzhou, China with IRB number 2023L20004/YZEDU/2023, and the data were obtained from the same hospital.

Pregnancy or other medical conditions, clinically significant immunodeficiency, evidence of infection, severe renal or hepatic disorder, and familial adenomatous polyposis or non-polyposis CRC were the exclusion criteria.

Keeping in view the above-mentioned criteria, a total of 57 patients were included in this study. After taking a comprehensive history, performing clinical examinations of the patients, and obtaining their informed consent, we determined whether they would take part in the study. Each clinicopathological characteristic was documented carefully. All the patients underwent a comprehensive observational and clinical evaluation before undergoing any kind of treatment. Patient characteristics (age and gender) and tumor features (e.g., tumor size, lymph node status, tumor location, metastases, and histopathological evaluation) were investigated. The American Joint Committee on Cancer (AJCC) categorization system was used to identify the clinical stages of cancer in people who were eligible for inclusion.

Oral administration of sulfate-free polyethylene glycol electrolyte powder was performed one day before the surgery to prepare the bowels for the procedure. The next step was to execute a standardized R0 surgical resection of colorectal carcinoma. All the surgical operations were carried out in a manner that was in complete accordance with the standards established by the National Ministry of Colorectal Cancer for the diagnosis and treatment of CRC. It was determined that the position of the carcinomas would determine which procedures were chosen. Laparotomy or laparoscopy was selected based on the findings obtained during the intraoperative procedure. After intestinal anastomosis, a peritoneal lavage, which is a crucial technique, was carried out using a solution of 1000 milliliters of 0.9% saline. This solution was then entirely absorbed. In the end, the specimen that had been removed was sent to a team of trained pathologists so that they could determine the TNM stage.

Raltitrexed was administered to all individuals. The dosage of raltitrexed was administered in accordance with the instructions provided by the manufacturer, which stated that it should be administered at 3 mg/m^2^ in a 0.9% NS injection in a volume of 500 milliliters. Following the closure of the abdominal incision or laparoscopic port, the solution was subsequently administered by means of a drainage tube into an abdominal cavity. It was a common practice to vibrate the belly sufficiently to ensure that the combined solution was distributed uniformly throughout the abdominal cavity to the greatest extent possible. The blended fluid was finally expelled from the abdominal cavity two hours after it had been administered. During this interim period, the drainage tube was sealed to avoid the leakage of chemotherapy medications from the abdominal cavity.

Each patient was noted for postoperative complications. This included fever, nausea/vomiting, pain abdomen, intra-abdominal bleeding, anastomosis leakage, abdominal cavity abscess, intestinal obstruction, wound infection, renal toxicity, hepatic toxicity, pulmonary infection, and deep vein thrombosis. No patient died during or after the procedure. The preformat form was created to incorporate all relevant factors specified earlier in the clinicopathological features. IBM SPSS Statistics for Windows, version 25.0 (released 2017, IBM Corp., Armonk, NY) was utilized in order to carry out the data analysis. Both qualitative and quantitative variables are provided as descriptive statistics. In this study, frequency and percentages were used to measure factors, such as gender, T-stage, lymph node status, metastasis, tumor clinical stage, pathological type, tumor location, and postoperative complications (fever, nausea/vomiting, pain abdomen, intra-abdominal bleeding, anastomosis leakage, abdominal cavity abscess, intestinal obstruction, wound infection, renal toxicity, hepatic toxicity, pulmonary infection, and deep vein thrombosis). For the quantitative variables, like age and average tumor size, the mean value was computed. According to common consensus, there is a significant link if p is less than 0.05.

Over the course of two years (December 2021 to April 2023), a prospective cohort study was conducted to investigate the correlation between various clinical and pathological features and the intraperitoneal chemotherapy drug raltitrexed. In just two years, the hospital received 132 reports of colon cancer cases; however, we employed a non-probabilistic sampling technique, and the final data analysis comprised 57 patients. The research findings were presented in accordance with the guidelines provided in the Strengthening the Reporting of Observational Studies in Epidemiology (STROBE) statement. This made sure that the data were fully and accurately understood.

## Results

A total of 57 patients participated in this study. Among them, there were 39 males constituting 69% of our study population and 18 females constituting 31% of our study population (Table 1).

**Table 1:** Demographic characteristics of the population

Variables		Frequency	Percent
Gender	Female	18	31.6
	Male	39	68.4
Disease stage	II	31	54.4
	III	26	45.6
Lymph node stars	No	31	54.4
	N1	16	28.1
	N2	10	17.5
T stage	T1	10	17.5
	T2	19	33.3
	T3	13	22.8
	T4	15	26.3
Tumor location	Right colon	18	31.6
	Left colon	16	28.1
	Rectal	23	40.4
Pathological type	Tubular adenocarcinoma	52	91.2
	Mixed adenocarcinoma	3	5.3
	Mucinous adenocarcinoma	2	3.5
Surgery-related complications			
Anastomosis leakage	No	55	96.5
	Yes	2	3.5
Intra-abdominal bleeding	No	56	98.2
	Yes	1	1.8
Deep vein thrombosis	No	56	98.2
	Yes	1	1.8
Raltitrexed-related complications			
Abdominal cavity abscess	No	54	94.7
	Yes	3	5.3
Wound infection	No	52	91.2
	Yes	5	8.8
Fever	No	40	70.2
	Yes	17	29.8
Nausea vomiting	No	36	63.2
	Yes	21	36.8
Pulmonary infection	No	55	96.5
	Yes	2	3.5
Pain abdomen	No	42	73.7
	Yes	15	26.3

Regarding disease stage, 31 individuals (54.4%) had stage II CRC, while 26 (45.6%) had stage III CRC. No lymph nodes were found in 31 individuals (54.4%), one lymph node was found in 16 individuals (28.1%), and two were found in 10 individuals (17.5%). For the tumor stage (T stage), 10 individuals (17.5%) had T1, 19 individuals (33.3%) had T2, 13 individuals (22.8%) had T3, and 15 individuals (26.3%) had T4. Regarding the tumor location, 18 individuals (31.6%) had a tumor in the right colon, 16 individuals (28.1%) in the left colon, and 23 individuals (40.4%) in the rectal area. For the pathological type, tubular adenocarcinoma was found in 52 individuals (91.2%), mixed adenocarcinoma in three individuals (5.3%), and mucinous adenocarcinoma in two individuals (3.5%). For surgery-related complications, anastomosis leakage was found in two individuals (3.5%), Intra-abdominal bleeding in one individual (1.8%), and deep vein thrombosis in one individual (1.8%).

Table 2 shows statistical measures related to factors associated with the development of complications. It is divided into two sections: significant factors for surgery-related complications and significant factors for raltitrexed-related complications (Table 2).

**Table 2:** Significant factors associated with the development of complications.

Complications		Frequency (%age)	Disease stage	Tumor location	Pathological type	T-stage
Significant factors for surgery-related complications						
Anastomosis leakage	No	55 (96.5)	0.709	0.653	0.015	0.369
	Yes	2 (3.5)				
Intra-abdominal bleeding	No	56 (98.2)	0.456	0.332	0.000	0.188
	Yes	1 (1.8)				
Deep vein thrombosis	No	56 (98.2)	0.456	0.332	0.000	0.188
	Yes	1 (1.8)				
Significant factors for raltitrexed-related complications						
Abdominal cavity abscess	No	54 (94.7)	0.433	0.280	0.001	0.094
	Yes	3 (5.3)				
Wound infection	No	52 (91.2)	0.585	0.115	0.029	0.394
	Yes	5 (8.8)				
Fever	No	40 (70.2)	0.332	0.785	0.806	0.215
	Yes	17 (29.8)				
Nausea/vomiting	No	36 (63.2)	0.126	0.044	0.084	0.383
	Yes	21 (36.8)				
Pulmonary infection	No	55 (96.5)	0.709	0.493	0.000	0.012
	Yes	2 (3.5)				
Abdominal pain	No	42 (73.7)	0.077	0.012	0.012	0.261
	Yes	15 (26.3)				

The columns "disease stage," "tumor location," "pathological type," and "T-stage" represent statistical measures (p-values) related to how different factors (disease stage, tumor location, pathological type, and T-stage) are associated with the occurrence of each complication.

For anastomosis leakage, the table provides information that 96.5% of the cases had no leakage, and 3.5% had leakage. The statistical values (e.g., 0.709, 0.653, 0.015, 0.369) indicate that the type of pathology is the only significant factor that determines leakage. Another positive association was T-stage with pulmonary infection and abdominal pain with the location of the tumor (p < 0.05).

Intra-abdominal bleeding, deep vein thrombosis, abdominal cavity abscess, wound infection, fever, nausea/vomiting, pulmonary infection, and abdominal pain are all significantly associated with the type of pathology present at the time of surgery.

**Figure 1:**
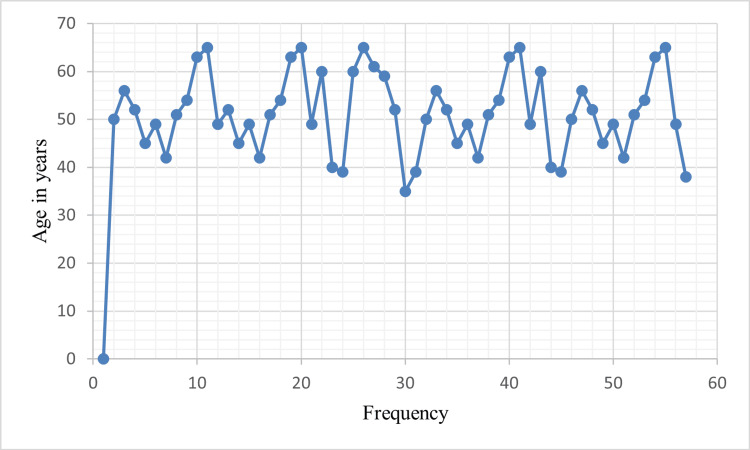
Age distribution in the study population

The age of the patients ranged between 35 and 65 years, and the average age was 51.29 years (Figure 1).

**Figure 2:**
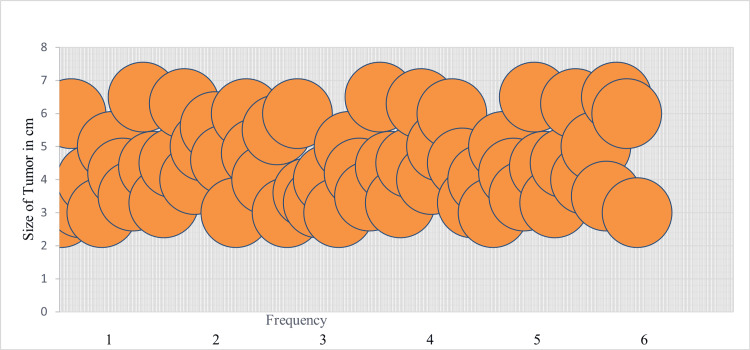
Average tumor size

The tumor size ranged from 3 cm to 6.5 cm, with an average tumor size of 4.48 cm (Figure 2).

**Figure 3:**
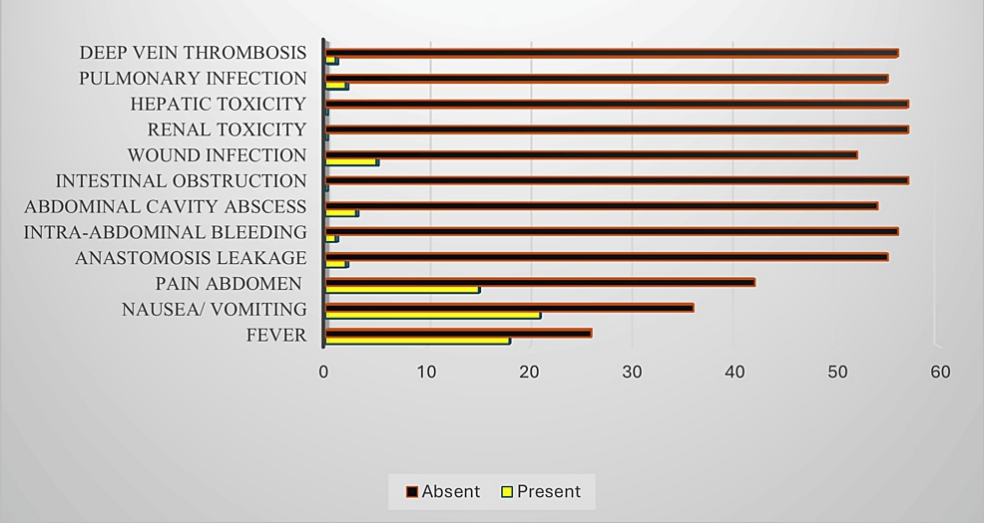
Frequency and percentage of postoperative complications

Fever, nausea/vomiting, and abdominal pain were the most common complications presented postoperatively (Figure 3).

## Discussion

Adjuvant treatment for patients with CRC currently involves the use of a fluoropyrimidine (5-fluorouracil (5-FU) or capecitabine) either alone or in conjunction with oxaliplatin for a duration of three or six months. Therapy selection is based on traditional histological staging methods, which are a crude method of patient classification. Patient stratification should make it possible to separate patients who have a good chance of recovering after surgery and a low risk of recurrence from those who have a higher risk of recurrence and would benefit more from chemotherapy in the long run. Artificial intelligence-driven models applied to digital photographs of removed tissue have revealed potentially helpful algorithms in the last few years that categorize patients into different prognosis groupings. Likewise, methods such as liquid biopsy, which gauges the amount of DNA from circulating tumors after surgery, can also be helpful in determining which patients have a high or low risk of their tumors returning [8].

Recently, the combination of chemotherapy and heat treatment for CRC was evaluated using organoids as in vitro models. The efficacy of chemotherapeutic drugs was tested in organoids at 37°C as a control and 43°C for 90 minutes to investigate the synergy of hyperthermia. Cell viability was measured using 10% CCK8. A library of CRC organoids using 22 patient parental tumors was successfully developed. The highest hyperthermia chemotherapy sensitization enhancement ratio (HCSER) score within each patient group revealed significant hyperthermia synergism in 11 of the 22 patient organoids that were raltitrexed, which means that hyperthermia enhanced the most effective effect of raltitrexed [9].

Fluoropyrimidine-based therapy used in metastatic CRC is the primary agent, but it can pose serious toxicities, especially cardiotoxicity, which is alarming [10].

Metastatic CRC relapse within the first two years after resection is between 60% and 70%. The MOSAIC (Multicenter International Study of Oxaliplatin/Fluorouracil/Leucovorin in the Adjuvant Treatment of Colon Cancer) study recommended adjuvant therapy of FOLFOX over 5-FU (fluoropyrimidine-based therapy). Adjuvant therapy is highly advised for high-risk patients. Resettable stage III colon cancer and 5-FU and oxaliplatin-based regimens are similar in their use, emphasizing the significance of systemic adjuvant therapy for most patients after resection of metastases [11].

The primary treatment for CRC remains 5-FU (extended infusion of 5-FU) while patients may endure discomfort because of the recommended extended infusion of 5-FU. Raltitrexed (Tomudex), a quinazoline derivative of folinic acid, is a direct and selective inhibitor of thymidylate syntheses (TS) with a convenient three-week dosing regimen. When severe cardiotoxicity or cardiovascular risk factors, gastrointestinal, and hematological toxicities become a substantial clinical issue necessitating cessation of fluoropyrimidine treatment, RTX can be considered a viable replacement. RTX is particularly useful for patients with a DPD deficiency, a critical gene involved in fluoropyrimidine metabolism that affects 3% of the general population. The majority of RTX-related deaths were caused by inadequate renal surveillance (creatinine clearance evaluation) or incorrect dehydration management linked with gastrointestinal toxicity, which reduced renal function. Otherwise, RTX’s ease of use contributed to the notion that it is a safe treatment [12].

In a trial of 86 individuals, 37.6% had previously experienced cardiotoxicity from fluoropyrimidine-based therapy. The overall and progression-free median survival times were 10.2 and 8.5 months, respectively. The side effects of raltitrexed were generally well-tolerated anemia that affected 41.7% of the patients, whereas nausea and vomiting brought on by chemotherapy affected 27.4% of the patients. Although 16.7% of the patients encountered grade 3, grade ½ toxicities predominated. Raltitrexed is a very effective and well-tolerated treatment for patients with CRC [10]. For patients with GI malignancies who have major CV toxicities or risk factors, a raltitrexed-based regimen is a well-tolerated therapy that is as effective as fluoropyrimidines. Patients on capecitabine (n = 110) and 5-FU (n = 45) had somewhat similar CV toxicity profiles. Of patients treated with raltitrexed, 13 (54%) developed cardiovascular toxicity and one (<0.15) passed away from myocardial infarction. The results showed that the median progression-free survival (PFS) was 36.0 months, while the median overall survival (OS) was 44.3 months [13].

IMRT combined with concurrent raltitrexed and irinotecan is a workable therapy option for recurrent CRC that is not curable. It provides an acceptable toxicity profile, good tumor response, and local control. IMRT (total dose: 50-60 GY in 25-30 portions) was administered on days 1 and 22 in parallel with irinotecan and raltitrexed (200 and 3 mg/m^2^, respectively) in 30 patients. A total of 17 patients showed stable disease, while 12 patients (40.0%) had an objective response. At the end of the follow-up, which had a median duration of 22 months; six (20.0%) patients had experienced local failure inside the irradiation field, four (13.3%) had experienced regional progression outside the irradiation field, 13 (43.3%) had experienced distant metastasis or metastatic progression, and nine (30.0%) had passed away. The local PFS (LPFS) was 23 months, and the median PFS was 13.5 months. Neutropenia (13.3%) was the most frequent grade 3 or 4 adverse event with a 26.7% occurrence [14].

In patients with advanced solid tumors, particularly malignant mesothelioma, and advanced CRC, combination chemotherapy has shown promising outcomes. Raltitrexed or Tomudex exhibits a single agent activity in a range of advanced solid tumors, but it has a different toxicity profile and a unique mode of action. In addition, the mechanism of action of raltitrexed differs entirely from that of irinotecan, oxaliplatin, and other medications with which it has been used. Preclinical evidence and these characteristics implied that raltitrexed in conjunction with 5-FU and other chemotherapeutic drugs or radiotherapy would lead to better treatments for a range of advanced solid tumors, including advanced CRC [15].

Patients in the raltitrexed/oxaliplatin group exhibited several notable differences from the 5-FU/leucovorin/oxaliplatin group, including a significantly higher partial response, disease control rate, and lower progressive disease. Severe anemia, asthenia, hepatic disorders, and nausea/vomiting were all significantly more common with the raltitrexed arm treatment. On the other hand, the 5-FU group experienced higher frequencies of grade ¾ alopecia and stomatitis/mucositis. In terms of overall response and OS, there were no statistically significant differences between the two regimens [16].

Tomudex has a therapeutic profile comparable to 5 FU and it is a well-tolerated drug. The possible renal toxicity can be avoided by adjusting the dose of the drug [17].

Raltitrexed-based doublets are associated with decreased rates of neutropenia, gastrointestinal toxicity, and rare cardiotoxicity as compared to earlier neutropenia, gastrointestinal toxicity, and rare cardiotoxicity. For advanced CRC, TOMOX and TOMIRI doublets are useful as first-line chemotherapy. For patients with cardiac comorbidities, dihydropyridine dehydrogenase insufficiency, or central line refusal, doublets represent a safe and efficacious therapeutic option [18].

The safety profile of first-line raltitrexed-based chemotherapy was satisfactory in patients with metastatic colorectal cancer (mCRC) who were not eligible for fluoropyrimidines. PFS and OS, regardless of related targeted treatments, were considerably superior in patients treated with TOMOX and in line with standard survival data in mCRC. When good prescription guidelines are followed, first-line raltitrexed-based chemotherapy with or without bevacizumab in patients with mCRC has an acceptable tolerability profile [19].

For patients with mCRC who are resistant or intolerant to conventional therapy, raltitrexed in combination with S-I and bevacizumab shows good antitumor effectiveness and safety, a favorable toxicity profile, and promising efficacy like the results of phase II trials. Patients with no peritoneal metastases, lower carcinoembryonic antigen levels, and no history of prior treatment with S-I and vascular endothelial growth factor inhibitors had a higher OS rate. There were no treatment-related deaths, and the overall incidence of adverse events was 88.6%, with mild to moderate AEs accounting for the majority [20].

Even with an early termination raltitrexed with oxaliplatin, combination therapy is safe and helpful for liver-only mCRC patients with chemoresistant disease [21].

The change of regimen from 5-FU to raltitrexed often is needed because of the intolerance with 5-FU. Raltitrexed also had a better three-year relapse-free survival [22]. Pressurized intraperitoneal aerosol chemotherapy (PIPAC) for patients with peritoneal metastases in a salvage scenario does not negatively impact their quality of life, showing stability in gastrointestinal symptoms and functional ratings, despite a transient increase in pain. However, the researchers caution against generalizing these results due to potential biases and the selective nature of the patient group, highlighting the need for further research to validate PIPAC's efficacy and impact on quality of life over time [23]. The results of our study also were similar to previous studies. Comprehensive genetic testing for all younger persons with CRC will direct treatment and increase our understanding of the pathogenic variations associated with this illness. Better detection of a family history of CRC can help further refine the risk profile of individuals who can benefit from early screening; risk prediction models that consider genetic risk, family history, and lifestyle and environmental factors might help identify additional individuals who might benefit from early screening. Lastly, trends in the incidence and mortality rates over the course of the following 10 years will offer empirical support for the new recommendations to start average-risk screening at age 45.

Limitations

The study involved a relatively small sample size of 57 patients. The patients were selected from a single center, so it may lack diversity. There is an inherent limitation due to the retrospective design of the study. The individuals with missing or incomplete data were not removed from the study as we already had a very small sample size. To include those patients with limited data, the variables of the study were reduced. Confounding variables could not be strictly addressed due to the retrospective design of the study.

## Conclusions

Raltitrexed is a potential treatment option since, as the clinical study on its use for intraoperative intraperitoneal chemotherapy in colon cancer patients demonstrates, it is not only dependable and effective but also has a favorable safety profile. Notably, the study's conclusions imply that raltitrexed can be taken as prescribed without causing severe hepatic, renal, or gastrointestinal toxicity concerns that are frequently associated with other chemotherapy medications. The most frequent side effect reported was nausea or vomiting, which is tolerable and does not lessen the treatment's overall beneficial effects. Because treatment safety and effectiveness are so important for better patient outcomes in colon cancer therapy, raltitrexed looks like a good alternative to standard systemic chemotherapy regimens. These preliminary findings are promising and call for additional research to confirm the long-term advantages and potential of raltitrexed in managing CRC throughout a broader range of stages of the illness and in conjunction with other therapy modalities.
